# 3D and organoid culture in research: physiology, hereditary genetic diseases and cancer

**DOI:** 10.1186/s13578-022-00775-w

**Published:** 2022-04-01

**Authors:** Elisa Suarez-Martinez, Irene Suazo-Sanchez, Manuel Celis-Romero, Amancio Carnero

**Affiliations:** 1 Instituto de Biomedicina de Sevilla, IBIS, Hospital Universitario Virgen del Rocío, Universidad de Sevilla, Consejo Superior de Investigaciones Científicas, Av Manuel Siurot sn, 41013 Sevilla, Spain; 2 CIBERONC, Instituto de Salud Carlos III, Madrid, Spain

**Keywords:** Organoid, 3D culturing, Stem cells, Disease modeling, Cancer

## Abstract

In nature, cells reside in tissues subject to complex cell–cell interactions, signals from extracellular molecules and niche soluble and mechanical signaling. These microenvironment interactions are responsible for cellular phenotypes and functions, especially in normal settings. However, in 2D cultures, where interactions are limited to the horizontal plane, cells are exposed uniformly to factors or drugs; therefore, this model does not reconstitute the interactions of a natural microenvironment. 3D culture systems more closely resemble the architectural and functional properties of in vivo tissues. In these 3D cultures, the cells are exposed to different concentrations of nutrients, growth factors, oxygen or cytotoxic agents depending on their localization and communication. The 3D architecture also differentially alters the physiological, biochemical, and biomechanical properties that can affect cell growth, cell survival, differentiation and morphogenesis, cell migration and EMT properties, mechanical responses and therapy resistance. This latter point may, in part, explain the failure of current therapies and affect drug discovery research. Organoids are a promising 3D culture system between 2D cultures and in vivo models that allow the manipulation of signaling pathways and genome editing of cells in a body-like environment but lack the many disadvantages of a living system. In this review, we will focus on the role of stem cells in the establishment of organoids and the possible therapeutic applications of this model, especially in the field of cancer research.

## Introduction

During the past decades, new ex vivo model systems that faithfully recapitulate human physiology in vivo have driven biological and biomedical research. Animal model systems are the closest to recapitulating body functions and cellular interactions in human tissues. Thus, they can predict how a treatment or a disease may develop, although they are limited by the differences among species biology, the differences in sensitivity, the cost of maintenance and a limited throughput [1, 2]. Two-dimensional (2D) monolayer cell cultures are more basic than animal models, but they provide insight into complex diseases with low cost and time required and high reproducibility in a way that proves to be both simple and efficient. However, this cell culture model places cells in a nonnatural environment without an extracellular matrix (ECM), which hinders the recapitulation of the complexity of the in vivo microenvironment and does not mimic the natural development of cells and tissues [3]. This deficiency has been observed in previous studies, for instance, by Sun et al. [4], whose studies on the sensitivities of human skin cells to dermatotoxic agents suggest that 2D cell culture is less likely to reflect physiological responses than three-dimensional (3D) models. Additionally, a different study showed that temozolomide resistance in glioblastoma 3D cultures was 50% higher than that in 2D models [5], which highlights the relevance of an ECM in cell culture studies.

**Fig. 1:**
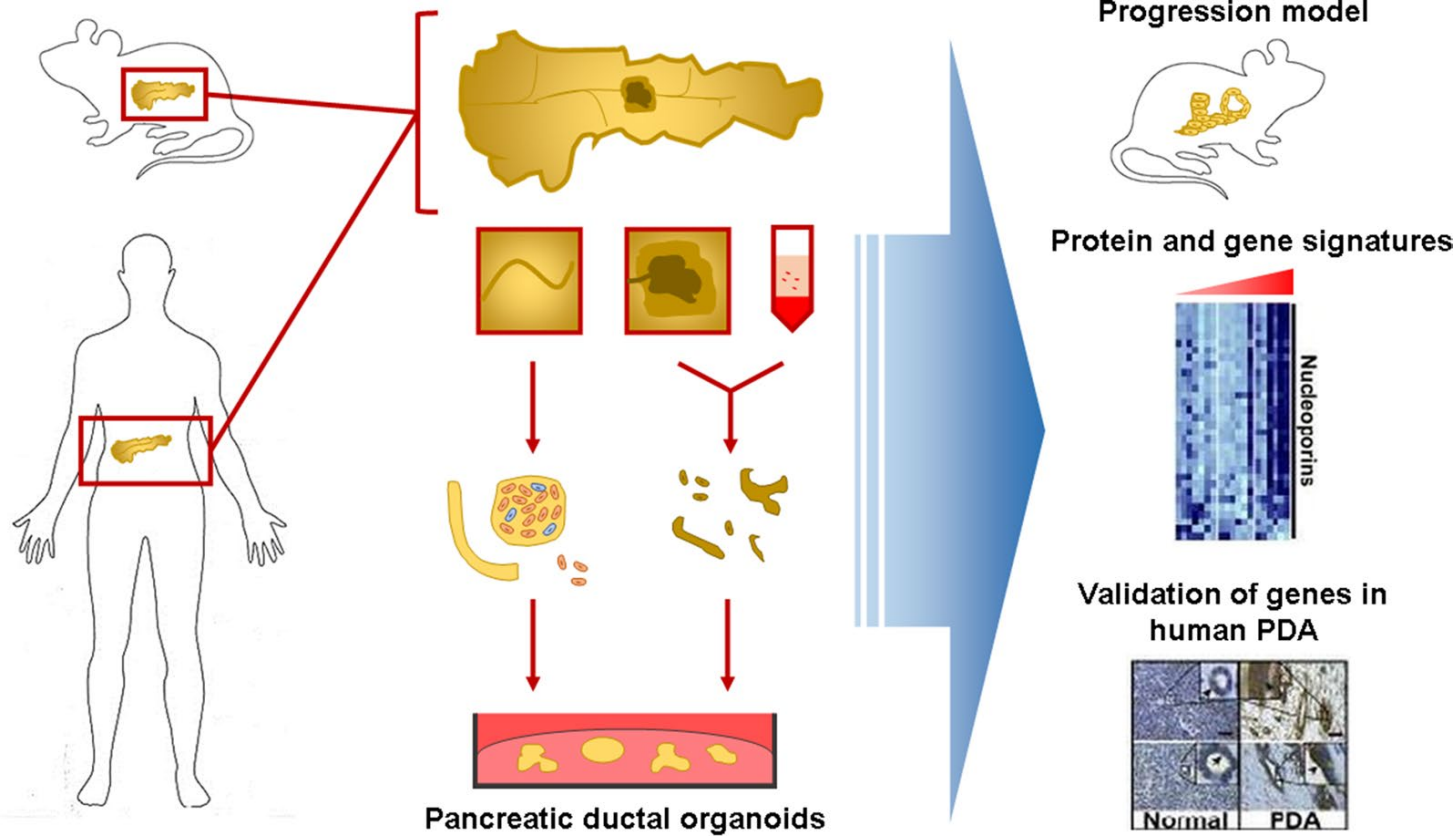
Generation of pancreatic ductal organoids from mouse and human pancreases and possible direct applications. Pancreatic tissue extract is digested and cultured to generate tissue-specific organoids. Organotypic cultures allow the creation of progression models in vivo, as well as the analysis and subsequent validation of proteins and genes involved in human tumor progression by phenotypic or histologic analysis

Due to the need for more accurate models, 3D culture technologies have been raised as a great alternative. Spheroids are a type of 3D cell structure formed by multicellular cell aggregates that better mimic cell–cell and cell–matrix interactions than 2D cultures, although they lack the capacity to recapitulate the tissue organization exhibited in vivo [6]. To achieve this complex organization, pluripotent stem cells (PSCs) appear to be a great tool because of their ability to differentiate into any cell type. When grown in a 3D matrix with specific growth factors and small molecule inhibitors, they grow into self-organizing organotypic structures, named organoids. Organoids can be defined as a collection of organ-specific cell types grown from stem cells that self-organizes through cell sorting and spatially restricted lineage commitment in a 3D structure recapitulating the process of self-organization during development in vivo and with functions similar to the organ that is being replicated [7] (Fig. 1).

Organoids can be obtained from various stem cells: tissue-derived adult stem cells (ASCs), embryonic stem cells (ESCs), induced pluripotent stem cells (iPSCs) and patient-derived tumor tissue cells. To display their potential, cells must be embedded in a specific ECM (usually Matrigel) with specific medium and niche factors. Medium components depend on the organoid model but mostly include HEPES, GlutaMAX, R-spondin-1 (Wnt agonist) and Wnt3A (Wnt signal activators, essential for leucine-rich repeat—containing G protein-receptor 5 (Lgr5^+^) stem cell development [8], and other growth factors such as epidermal growth factor (EGF), fibroblast growth factor (FGF), nicotinamide, Noggin (bone morphogenetic protein (BMP) inhibitor), B27, small molecule inhibitors such as Y27632 (Rho kinase inhibitor), and hormones that allow organoid growth. Organoids can be expanded long term, genetically modified, and cryopreserved, maintaining their phenotypic characteristics [9, 10].

In this review, we discuss the developmental process in organoid formation, the current use of organoids in biomedical and cancer research, the relevance of the microenvironment in organoid formation and the potential applications of this 3D model in drug development and personalized medicine.

## Establishment of organoids

### Stem cells in organoids

Stem cells (SCs) are undifferentiated or partially differentiated cells that are capable of long-term self-renewal and can produce cells of different lineages. These cells are the source of all the structures in our bodies and are responsible for the formation and homeostasis of adult tissues [11]. There are pluripotent stem cells (PSCs) and adult stem cells (ASCs), which share some common characteristics but also show different traits. Pluripotent cells are able to differentiate into all cell types present in an embryo or an adult, while adult stem cells can only differentiate into cells from the organ in which they originated [12]; thus, they are called multipotent or unipotent cells. While ASCs are present in organs and tissues throughout the majority of postnatal life, PSCs are only present naturally in embryos; thus, they are called embryonic stem cells (ESCs). However, they can be artificially obtained through the dedifferentiation of other cells using recently developed methods. The first to obtain this kind of cell, called an induced pluripotent stem cell (iPSC), was Takahashi and Yamanaka in 2006, due to the ectopic expression of four transcription factors (OCT4, SOX2, KLF4 and MYC, as OSKM) in mouse fibroblasts [13]. Since then, we have been able to obtain human iPSCs derived from various cell types and to substitute some of those transcription factors with small molecules that also allow for the reprogramming of cells [14, 15]. However, there is still much research to do to fully understand this process and discover optimal reprogramming conditions.

To create an organoid, it is necessary to have a stem progenitor, either a PSC or an ASC. This progenitor can be a single cell, in the case of ASCs, or an aggregate of cells, as in PSC-derived organoids. PSCs need an intermediate state to generate an organoid called an embryoid body (EB), which is composed of a 3D aggregate of pluripotent stem cells [16]. Multiple organoid models have been developed using both types of stem cells, which are more or less suitable for different organoid types depending on their characteristics and embryonic origin.

### Adult stem cells in organoids

ASCs are responsible for maintaining the homeostasis of body tissues through self-renewal and differentiation. This kind of stem cell has been found in several tissues, for instance, neural SCs (NSCs) in the brain [17, 18], hematopoietic SCs (HSCs) in the bone marrow [19, 20], hepatic progenitor cells in the liver [21, 22], mesenchymal SCs in adipose tissue [23], crypt SCs in the intestine [24, 25], epithelial SCs in the skin [26], germline SCs in the seminiferous tubules [27], and muscle SCs in myofibers [28]. Their self-renewal is regulated by many different signaling pathways, including the Wnt, Sonic Hedgehog (SHH), Notch and phosphatidylinositol 3-kinase (PI3K) pathways, together with polycomb proteins [29]. They are embedded in a specific microenvironment called the stem cell niche, which regulates their cellular fate through secreted molecules, cell–cell interactions and physical contact with other cells and the extracellular matrix [30]. In normal tissues, ASCs are usually in a quiescent state, in which they do not replicate unless they are needed to maintain homeostasis or repair the tissue. This process is regulated by cellular mechanisms and external signals from their microenvironment [31, 32].

ASCs and their progeny undergo self-organization processes influenced by their niches, which can be mimicked in vitro under specific culture conditions. Due to their origin, organoids generated from ASCs or adult tissue fragments more closely resemble the homeostatic and regenerative capacity of the tissue of origin than PSCs [33]. For that reason, they are a good model to study diseases, such as cancer or neurodegenerative disorders [9]. They have also been demonstrated to be more genetically stable than their PSC counterparts [34, 35].

Since the first organoid was established in 2009 from intestinal stem cells [8], organoids from several tissues have been developed from ASCs, including stomach, liver, pancreas, prostate, mammary gland, fallopian tubes, taste buds, lungs, salivary glands, esophagus [9], epididymis [36], lingua [37], lacrimal gland [38], and thyroid organoids [39] (Fig. 1). However, obtaining organoids from ASCs can be a challenge due to the need to obtain samples from the organ of origin of the stem cells and their restricted differentiation capacity. These issues have been addressed by using PSCs, which can be easily obtained from fibroblasts from a patient and expanded, therefore generating a model without time or availability limitations.

### Pluripotent stem cells in organoids

In recent years, there has been intensive study in the field of PSCs. These cells are known for their self-renewal capacity and their ability to produce differentiated cells from tissues originating from the three germ layers (ectoderm, mesoderm and endoderm). Due to ethical concerns regarding the use of human ESCs, the development of iPSCs has translated into an important advancement in the generation of organoids because they are easy to obtain, highly reproducible, and can generate multiple tissue types. Nevertheless, in comparison with ASCs, they produce a kind of organoid that more closely resembles the fetal tissue stage [33]. Therefore, they can be exploited as a model for developmental processes and organogenesis and their associated diseases.

- iPSCs
  If they are exposed to the right combination of growth factors and signals and provided with a 3D scaffold, iPSCs can start differentiating into different cell types and self-organizing into organoids. With this approach, organoids resembling multiple tissues have been successfully developed, such as the brain [40], eyes [41], kidney [42], lung [43], stomach [44], intestine [45, 46], inner ear [47, 48], skin [49, 50], thyroid [51] and liver [52].
  iPSC-derived intestine organoids have been shown to contain functional enterocytes, goblet cells, Paneth cells and neuroendocrine cells [45], and gastric organoids present both the antrum and corpus compartments [44]. Lung organoids have exhibited a relevant similarity to the native organ since they show both mesenchymal and epithelial components [53]. Kidney organoids also contain all the expected cell populations (glomerulus, proximal tubule, loop of Henle, distal tubule, collecting duct, endothelial system, and the interstitium), although their transcriptional profile is more similar to the first trimester kidney than to the adult kidney [54–56]. Organoids of various regions of the brain have been cocultured and generate a dorsal–ventral axis [57] and allow the analysis of interneuron migration and integration during development [58].
  Altogether, these achievements suggest that iPSC-derived organoids represent a very promising tool. However, generating 3D models from iPSCs that fully recapitulate human physiology remains a challenge. One of the main problems with this type of organoid is finding a way to provide all the cells with oxygen and nutrients and to remove the waste substances. The lack of a vascular system only allows for the growth of small organoids (micrometers to millimeters in scale) because it relies on the diffusion of nutrients. This often leads to necrosis of the inner core of the organoid [59]. However, multiple options have been proposed to overcome this issue. The two main strategies available to recreate an in vitro vascular circulation would be to create new blood vessels within the organoid through the seeding of endothelial cells or to use a synthetic scaffold. Most of the systems currently being used integrate both approaches and some sort of microfluidic bioreactor to keep the fluids circulating among the cells [60–62]. A spinning bioreactor, whose spinning enhances nutrient and oxygen availability, has also been successfully used to improve brain [63], kidney [64] and retinal organoids [65].
  Other challenges to overcome in this type of organoid are the heterogeneity and the lack of maturation that they present. Due to the stochastic nature of the self-differentiation process and the complex and long protocols needed to produce organoids from iPSCs, they show significant batch-to-batch differences. Furthermore, the short lifespan of these systems does not allow for the total maturation of cells, leading to a fetal-like phenotype [66]. In conclusion, this type of organoid still has some issues that need to be addressed before it becomes a widely employed technique, both in basic and applied research. Nevertheless, they are already being successfully used to study some processes and diseases, and we expect them to be included in the experimental design of more investigations in the near future.
- ESCs
  ESCs are pluripotent cells that exist in embryos during the first days post-fertilization. They have the ability to divide indefinitely and to originate every other cell of the organism [67]. This kind of cell can also originate from organoids; however, their differentiation into a specific tissue is a complicated process. They require a long timespan and different cocktails of growth factors to generate these structures because the system needs to mimic embryonic development [9, 68]. They also raise considerable ethical concerns, especially in the case of human ESCs, because the method to obtain them is via the destruction of viable embryos to obtain cells before 3 months of gestational age [69]. Some alternatives have been proposed, for instance, obtaining cells from earlier stages of development to preserve the embryo and allow it to grow [70, 71]. Nevertheless, this approach exhibits its own problems, such as the need for a feeder layer to establish a functional hESC line [72, 73] or an extracellular matrix or a 3D culture model [74, 75].
  Despite these problems, ESCs constitute quite satisfying models for development, genetic, and infectious disease research, especially for tissues with low regenerative capacity. Moreover, ESC-derived organoids show high complexity because they can include mesenchymal and endothelial components, in addition to the epithelial component that ASC-derived organoids normally present [9]. ESCs have been successfully differentiated into lung organoids, which include epithelial and mesenchymal cells and are able to produce surfactant [43]. They have also generated islet organoids of the pancreas, with functional alpha, beta, delta, and polypeptide cells, with the ability to secrete insulin-secretory granules [76, 77]. Organoids of different regions of the brain have also been generated, for instance, mid-brain-like organoids [78] and cortical-like organoids [79]. Recently, Cakir et al. developed a system to provide cortical-like organoids with a complex vascular-like network, which allowed them to have better functional maturation [80]. In addition, organoids from other tissues have been generated from ESCs, including hepatic, prostate, thymic, kidney, thyroid, stomach, heart, inner ear, salivary gland, and skin organoids [81] (Fig. 1).
  In conclusion, the different types of stem cells present their own advantages and inconveniences for the generation of organoids. To generate a specific organoid, it is necessary to take into consideration its tissue of origin, as well as the type of model needed for the study, to decide which type of cell is best suited for the purposes. Below, we describe the state of the art of established human organoid methods derived from both PSCs and ASCs.

### Organoids of different nontumoral tissues

#### Small intestinal and stomach organoids

The small intestinal epithelium is composed of a set of crypts with differentiated cell types. Lgr5^+^ stem cells are located at the bottom of crypts and are controlled by different signaling pathways. For instance, Wnt and Notch are key to maintaining stem cells in an undifferentiated state and driving proliferation. EGF has a mitogenic effect, and BMP signaling is a negative regulator of crypts; thus, when Noggin inhibits the BMP pathway, it results in a suitable environment for crypt formation [8].

In 2009, Sato and colleagues developed the first long-term stable intestinal organoid from mouse small intestinal crypt Lgr5^+^ stem cells based on the high self-renewal capacity of stem cells in the intestinal epithelium [82]. This new culture method implied a major technological advance for this field, although it is relatively simple. They used a suspension of Lgr5^+^ single cells or crypts in Matrigel supplemented with growth factors simulating niche signals during repair, such as Wnt, R-spondin-1, EGF and Noggin, to promote and maintain cell proliferation. The resulting crypts simultaneously generated villus-like epithelial structures containing all cell types, faithfully recapitulating, almost physiologically, the intestinal structures from the tissue of origin. In another study, researchers found a connection between CD24^+^ Paneth cells in the small intestine and Lgr5^+^ stem cells [83]. Coculture with CD24^+^ Paneth cells provides the niche factors required for Lgr5^+^ stem cell maintenance, which improves organoid formation. Nevertheless, exogenous Wnt can substitute for Paneth cells.

The addition of Wnt3A to intestinal organoid culture along with other growth factors allows the infinite expansion of mouse colon crypts [84]. Moreover, the addition of nicotinamide, A83-01 [a selective TGFβ type I receptor kinase (ALK) inhibitor] and SB202190 (a p38 inhibitor) was required for the long-term growth expansion of both human small and colon organoids. In a different approach, iPSC-derived intestinal organoids can be established by differentiating iPSCs into definitive endoderm by using activin A and the addition of Wnt3A and FGF2, which can further differentiate into hindgut endoderm or into posterior foregut. iPSC-derived organoids harbor niche factor-producing mesenchymal cells, so unlike ASC-derived organoids, they need fewer niche factor requirements [85].

Related to the methods described above, stomach organoids are generated in a similar way due to their resemblance to the intestinal epithelium. The gastric epithelium presents Lgr5^+^ stem cells at the base of pyloric glands responsible for the self-renewal of the tissue [86] that are able to generate long-term organoids [87], as well as those generated from PSCs with success [88]. In addition, gastric organoids can also be established long-term from isolated TROY^+^ chief cells [89, 90], a multipotent stem cell type in the murine gastric corpus.

#### Pancreatic and liver organoids

Both the pancreas and liver come from endoderm progenitor cells in the foregut during embryogenesis, resulting in similarities in the function and morphology of these mature organs. Despite sharing similarities, adult organs have different cellular functions and regenerative capacities; in this sense, the liver has much more regenerative capacity than the pancreas [91, 92].

In the pancreas and liver epithelia, *Lgr5* is not expressed in progenitor duct cells until there is tissue damage, so Lgr5^+^ duct cells recovered from damaged tissue would have the ability to form organoids in a culture with specific niche factors. It has been proven previously that Lgr5^+^ cells from injured mouse liver [93] and mouse pancreas [94] form the respective organoids with self-organization and long-term expansion of ductal progenitor cells (Fig. 1). Liver progenitor cells are cultured in Matrigel as ECM and medium with growth factors such as hepatocyte growth factor (HGF), FGF, EGF and R-spondin-1 to mimic the in vivo physiology in the development of liver organoids [35]. In a more recent study, Hu et al. grew long-term functional mouse and human liver organoids using Wnt and HGF signaling. Later, these organoids were successfully engrafted in mouse models recapitulating the proliferative response of hepatocytes after tissue damage [95].

However, Georgakopoulos and Prior et al. [96] developed a method to expand human pancreatic organoids derived from donor tissue with high efficiency by the addition of a TGFβ inhibitor, forskolin, prostaglandin E2 and an increased concentration of R-spondin-1 to the organoid medium. Human pancreatic organoids recapitulated the morphology, epithelial polarization and genomic stability of their tissue of origin (Fig. 1). However, this method is unable to generate organoids from single cells, which would help in disease modeling of the exocrine compartment and potential cell therapies.

#### Lung organoids

3D lung models can be derived from different progenitor cells, such as basal cells in the mucociliary epithelium, Clara cells in the airway epithelium and specialized alveolar type II cells (AEC2s), found with specialized alveolar type I (AEC1s) cells in the alveolar sacs [97].

The first lung organoid [98], called the ‘tracheosphere’, was derived from mouse and human basal cells. The protocol consisted of isolating tracheal basal cells and embedding them in Matrigel and MTEC/Plus medium [99], described before for in vitro studies of mouse tracheal epithelial cells. The ‘tracheospheres’ formed were clonal and contained both secretory and ciliated cells, but basal cells lost the ability to differentiate after several passages. Another study using this model proved the relevance of IL-6/STAT3 signaling in the differentiation of basal cells [100]. Stat3 inhibitors and IL-6 reduced the generation of ciliated versus secretory cells since Notch signaling is required for the differentiation of basal cells toward the secretory type [101]. AEC2-derived organoids, called ‘alveolospheres’, contain both AEC2 and AEC1 present in the alveoli but are not yet fully defined and need the presence of lung stromal cells for growth and differentiation [102]. These organoids cannot yet faithfully recapitulate the complex alveolar region or other structures and cellular interactions of the lung.

#### Brain organoids

Current brain organoid protocols are limited given the high complexity of the human brain, both structurally and functionally. However, they replicate some of the key physiological features of the brain during organogenesis. Most protocols are based on the optimization of neural induction from iPSCs or ESCs by mimicking endogenous features in vitro [59, 60, 103, 104]. Neural induction requires the inhibition of SMAD signaling to promote ectoderm formation [105] and inhibition or activation of Wnt signaling depending on the region trying to be replicated; for example, for forebrain organoids, Wnt activation is necessary [60, 106, 107], whereas inhibition of Wnt signaling promotes anterior brain differentiation [108, 109]. However, the effect of Wnt signaling and other growth factors has not yet been fully explored, given the complexity of the interactions found in vivo in the brain regions.

Recently, a study carried out in 2018 showed a method for transplanting human brain organoids into adult mouse brains to provide a vascularized and functional environment, triggered by the in vitro limitations in synaptic connectivity and interactions with the immune system [110]. These organoids recapitulate late embryonic or early postnatal tissue, and only the one that undergoes vascularization survives, integrating and forming functional circuits within the mouse brain. A similar approach with patient iPSC-derived organoids would be interesting in the treatment or study of complex brain disorders.

#### Retinal organoids

Most vision impairment and blindness are related to retinal degeneration diseases and photoreceptor (rod and cone) damage, without a definite cure known to date. The development of PSC and iPSC technology in the establishment of organoids has opened a new research field with a high potential in retinal tissue modeling and repair in patients [111]. Current protocols are based on Sassai and colleagues’ works in retinal development in vitro from mouse ESCs (mESCs) [112, 113]. The protocol consists of a reaggregation of mESCs embedded in Matrigel, which leads to optic vesicle-like structures within the first week of culture. Optic vesicle-like structures change shape by invagination, forming optic cup-like structures that will develop into a stratified neural retina containing an outer nuclear cell layer, an inner nuclear cell layer and a ganglion cell layer with a similar apical-basal polarity found in vivo.

Recently, retinal organoids were generated from human iPSCs (hiPSCs) mimicking the human retina in a physiological manner [114]. Photoreceptors contained in hiPSC-derived organoids were found to react to light stimuli and to pass the information to the inner retina layer, similar to in vivo observations [115, 116]. In a study carried out in 2020 by Mei-Ling et al. [117], the first late-onset retinitis pigmentosa patient iPSC-derived retinal organoid was generated with a consistent phenotype. This study provides new insights into the mechanism of complex retinal diseases, which still requires further exploration.

### Relevance of the microenvironment in organoid development

**Fig. 2:**
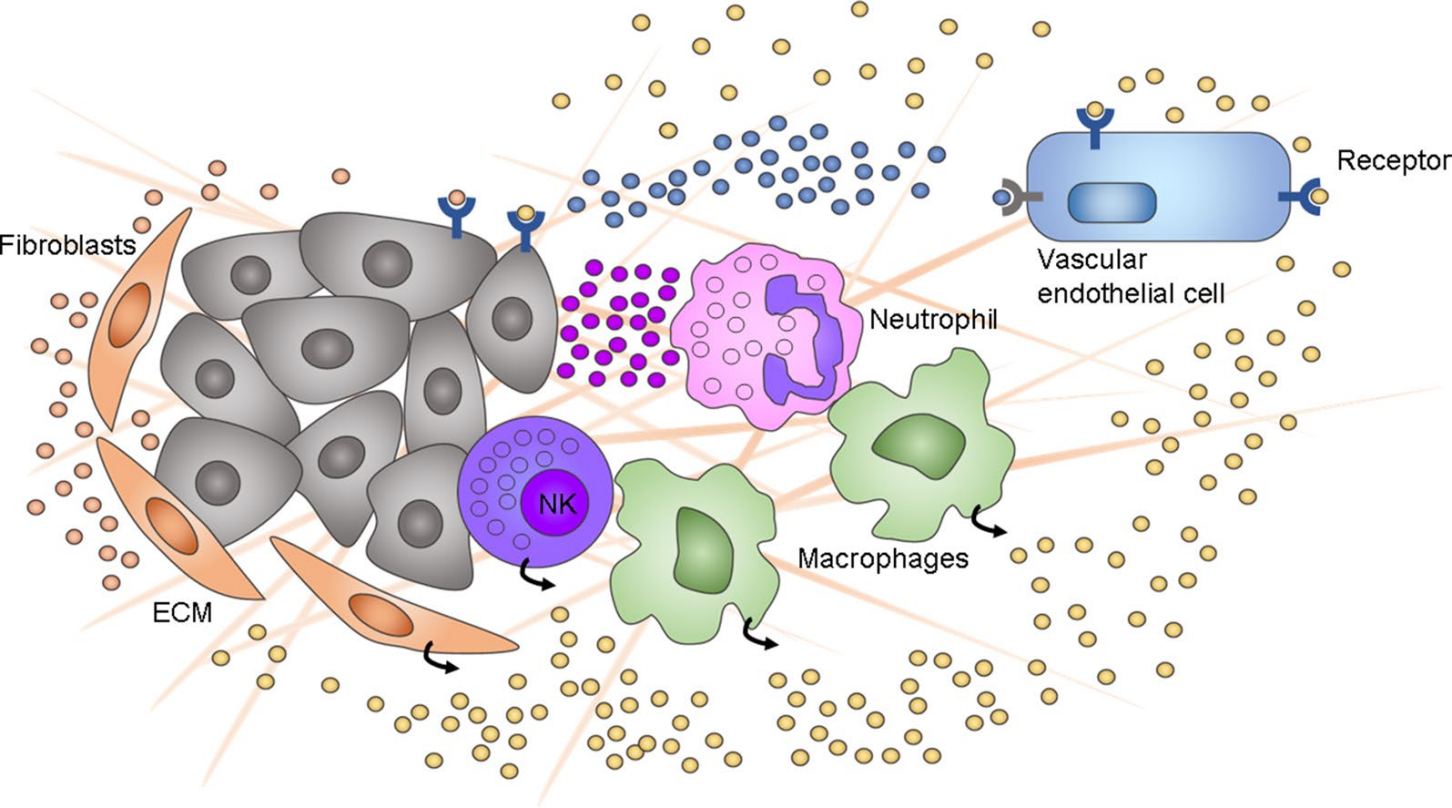
Schematic view of the multilineage nature of the tumor microenvironment. Fibroblasts, NK cells and macrophages adjacent to cancer cells secrete cytokines and other signaling molecules that stimulate vascular endothelial cells and activate immune cells, which in response attack tumor antigen-displaying cancer cells

The tissue microenvironment is generally constituted by diverse and complex physical/chemical interactions among multiple tissue-specific cell types, stem cells, immune system cells, stromal cells, several soluble factors and the ECM. The microenvironment influences the phenotypic outcomes of cells in both healthy and damaged tissue [118] and is implicated in the maintenance of stem cells [119, 120], progression of cancer [119, 121, 122] and gut cell–microbiota interactions [123]. Thus, in organoid models, the in vitro microenvironment must be optimized as much as possible to obtain the most accurate results, especially in those studies where cell interactions with their surroundings are a key factor in the evolution of the model (Fig. 2).

The study of the microbiota as a great part of the intestinal epithelium microenvironment can help to understand the interactions between them. The host microbiota is suggested to regulate molecular and cellular mechanisms by interacting with receptors on the surface of host cells and producing metabolic products that modify host physiology and the immune system response [123, 124]. When the intestinal microenvironment is altered, several diseases, such as inflammatory bowel disease [125] and obesity [126], can appear. Various studies have combined the microbiota with organoid technology. *Lactobacillus reuteri* D8, a commensal bacterium in the small intestine, was cocultured with lamina propria lymphocytes, and intestinal organoids exhibited a protective effect by promoting the proliferation of intestinal epithelial stem cells through the activation of STAT3 signaling [127]. This effect was also observed by activation of the Wnt/β-catenin pathway in intestinal organoids cultured in Matrigel [128]. Moreover, Hill et al*.* microinjected a nonpathogenic *Escherichia coli* strain, ECOR2, into the lumen of hPSC-derived intestinal organoids to define an in vitro model for the neonatal intestine, and this association resulted in enhanced epithelial barrier function and integrity [129].

In addition, the study of the tumor microenvironment (TME) has become an important factor in cancer research due to the increased possibility that the development of the TME is critical to the continued uncontrolled proliferation of cancer cells (Fig. 2). Neal et al. [122] cocultured patient-derived organoids from human donors and mice with native immune cells using the air–liquid interface (ALI) organoid method. The organoids preserved the original tumor T cell receptor spectrum and successfully modeled immune checkpoint blockade. Another ALI culture system incorporates peripheral and tumor-derived immune cells from the patient into patient-derived tumor organoids to mimic the immunosuppressive TME to analyze the tumor response to the patient’s therapy [130].

## Therapeutic applications

### Cancer

#### Cancer stem cells in organoids

Similar to what happens in normal tissues, it has been proposed that tumors have some cells responsible for the generation and maintenance of the rest of the cells [30]. In the context of cancer, they are called cancer stem cells (CSCs or TICs for tumor-initiating cells), and they share common characteristics with their healthy counterparts. For instance, they show indefinite self-renewal, and they can produce different types of cells through asymmetrical division. However, they show an aberrant response to intrinsic and extrinsic signals that regulate the fate of stem cells in normal tissues. They have been described as the initiators of tumors and are needed for tumor progression and for the colonization of metastatic niches [131]. These CSCs can also shift between a proliferative and a quiescent state, the latter being linked to their ability to escape anticancer therapy and to attack the immune system [132, 133]. The crosstalk with their niche, composed of cells residing in the tissues, immune cells, their extracellular matrix and signaling molecules, is also crucial for CSCs. Their properties can be altered by interactions with their microenvironment, and processes such as inflammation, hypoxia or wound healing might favor carcinogenesis. Additionally, CSCs may also change their environment in their favor [134–136].

The CSC hierarchical model proposes that this subpopulation of cells can self-renew and produce intermediate progenitors, which subsequently generate terminally differentiated cells, thereby giving rise to the heterogeneity of tumors [137]. There is significant evidence supporting this hypothesis; for instance, stem cells have been found to be the origin of tumorigenesis in colon cancer [138, 139], basal cell carcinoma [140] and some brain [141, 142] and breast tumors [143]. However, it has been demonstrated that this hierarchy is not always static and that there is a certain plasticity between progenitors and CSCs. The dedifferentiation of tumor bulk cells to CSCs has been described in breast [144, 145], lung [146, 147], melanoma [148, 149], ovarian [150], glioma [151], pancreas [152], and colon [153–155] cancer. This plasticity suggests that differentiated cells can acquire stem cell properties and vice versa, depending on the interaction of the cells with their microenvironment and different stressors [137]. Dedifferentiation might occur through different mechanisms, including epithelial to mesenchymal transition (EMT) and expression of Yamanaka factors, cell cycle activators or other developmental genes [156].

**Fig. 3:**
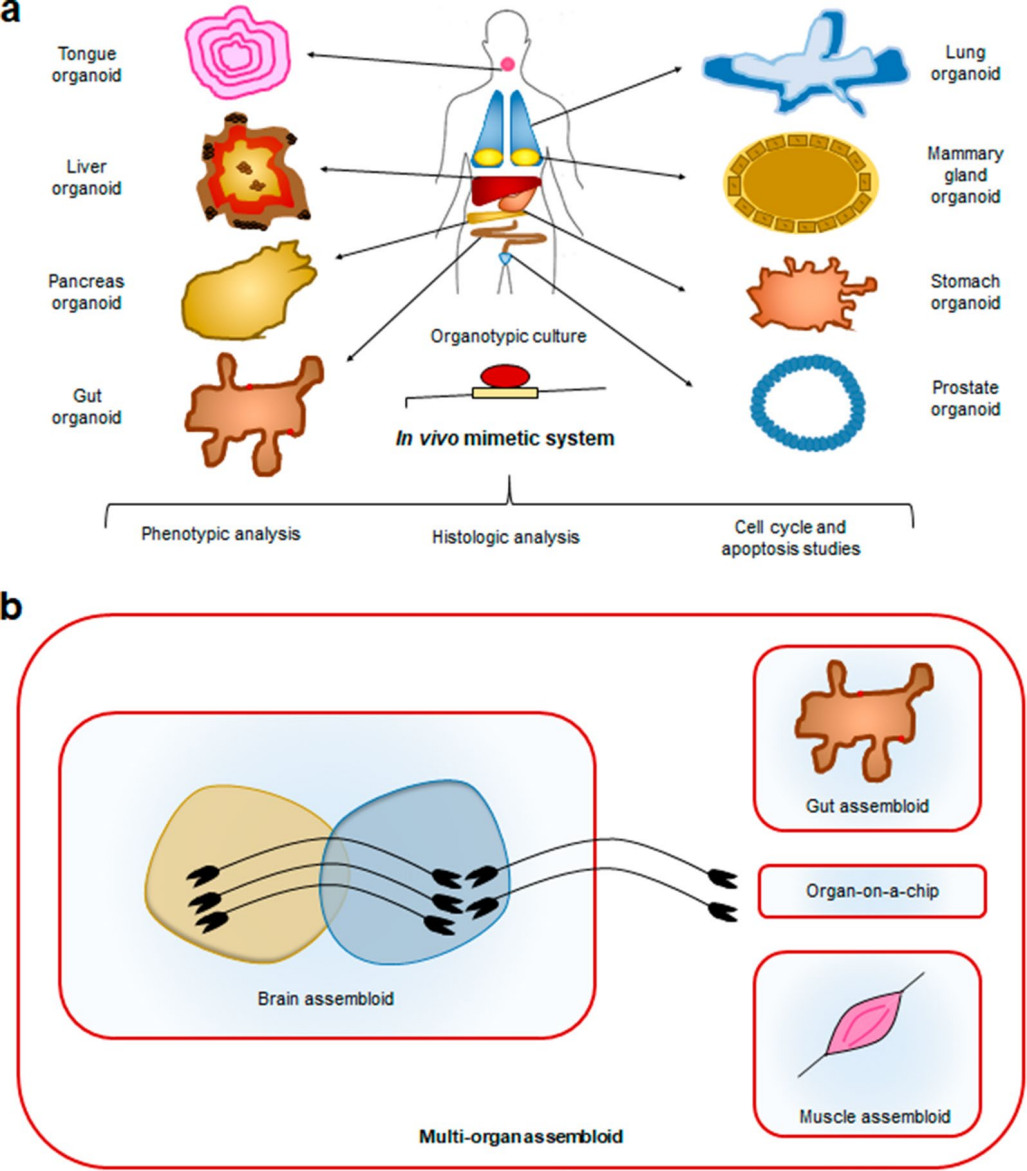
**a** Tissue-specific organoids generated from human tissue. The mimicry of in vivo conditions produced by organotypic cultures allows for different types of cellular and tissue studies. **b** Optimization of organoid culture techniques permits their combination and the creation of multilineage or multiorgan assembloids that facilitate the in vitro study of many more complex organ and wider body systems. Multiorgan systems may further benefit from the incorporation of emerging organ-on-a-chip approaches, an emerging technology that places biomimetic cultures in a microfluidic chip allowing the simulation of multiple organ environments at a microscale

Because of their contribution to cancer progression and metastasis, therapeutic resistance, tumor recurrence and evasion of the immune system, notable efforts have been made to investigate CSC mechanisms of action and to design better therapies specifically targeted to them. With this purpose, numerous models have been proposed that recapitulate the tumor characteristics and heterogeneity to different extents (Fig. 3A). In the context of cancer research, in addition to organoids, other types of 3D models are very popular. For instance, spherical cancer models are the most commonly used 3D in vitro model due to their easy production [157]. They are composed totally or partially of cancer cells, and they all share a spherical shape. Different types of spherical models have been developed, with diverse properties and suitability for different uses, that were classified into 4 main categories by Weiswald et al. [158].

Tumorspheres are a model of CSC expansion that originate by cultivating tissue-derived cells or established cell lines in low-attachment conditions after seeding at a low density to avoid aggregation. This model allows for the clonal expansion of the CSCs that are present in those cultures, maintaining their multipotency and 3D interactions and even showing a gradient of oxygen and nutrients from the core to the outer layers (Fig. 3A). However, this model lacks nonneoplastic cells; therefore, it does not support the study of tumoral cell interactions with their niches, and it is usually employed to specifically study CSC properties [159]. Multicellular tumor spheroids (MCTSs) are generated by seeding an elevated number of cells, typically from an established cancer cell line, under nonadherent conditions and stimulating their aggregation and compaction. This type of model can include tumoral and nontumoral cells if they are cocultured, and the cells form very packaged structures, including intermediate junctions between them [160, 161].

Organotypic multicellular spheroids (OMSs) and tissue-derived tumorspheres were both obtained from patient tumor fragments, but in the first case, they were nondissociated, and in the second case, they were partially dissociated and remodeled and compacted later (Fig. 3B). Thus, OMSs are able to maintain elements of the stroma, such as immune cells and extracellular matrix, for up to 70 days after their establishment, while tissue-derived tumorspheres are exclusively composed of tumoral cells [162, 163].

Although spherical cancer models are useful, they fail to fully recapitulate the real conditions of a tumor in a living organism; thus, organoids have arisen as a promising near-physiological model in cancer research due to their potential to represent the tumor microenvironment at the level of individual patients, which makes this technology a great tool in the personalized medicine approach. Cancer organoids or tumoroids have been successfully established from both ASCs and PSCs, including for the prostate [164], breast [165, 166], pancreas [167–169], liver [170, 171], ovary [172], esophagus [173, 174], colon [82, 175], stomach [176] and fallopian tubes [177].

Cancer organoid cultures enable the maintenance of interpatient and intratumor variations derived from different parts of the primary tumor or from metastatic cells, representing intratumor or intertumor heterogeneity at any stage [178]. Intratumor heterogeneity is represented by tumor cells that harbor single unstable genomes that might contribute to therapy resistance and cancer progression [34]. Patient-derived organoids (PDOs) from different regions of the same tumor retain the heterogeneous genetic composition [166, 173, 176]; however, they need to be characterized in-depth before experimental procedures due to the possible different sensitivities of tumor subclones to the same treatment and the acquisition of novel mutational signatures over time. Surprisingly, tumor organoid growth does not surpass that of normal organoids; consequently, epithelial cells from the remaining healthy tissue of a biopsy can overgrow tumoral cells. Therefore, it is vital to properly isolate the tumoral material or to cultivate the cells under selective media that only allows for the expansion of cancerous cells [179, 180]. Additionally, tumor organoids show the same issues as normal organoids, for instance, the lack of stroma, blood vessels and immune cells and thus do not fully recapitulate the real tumor. Therefore, it would be interesting to investigate how to generate more complex organoids with the presence of those components.

However, the high efficiency in organoid culture from patient-derived tumor cells and matching healthy cells has enabled the generation of highly characterized PDO biobanks that make these resources available to the research community. Several initiatives have been developed, such as the nonprofit company Hubrecht Organoid Technology (HUB) (https://huborganoids.nl/) in The Netherlands, which biobanks and distributes well characterized PDOs representing a variety of organs and disease models, and The Human Cancer Models Initiative (HCMI) (https://ocg.cancer.gov/programs/HCMI). The HCMI is a collaborative international consortium created by the US National Cancer Institute (NCI), Cancer Research UK (CRUK), the Wellcome Sanger Institute (WSI), and the foundation HUB that generates tumor-derived culture models and aims to provide a resource of novel characterized cancer models to the research community with clinical and molecular data.

Due to the characteristics that they are able to mimic, tumor organoids obtained from patients are very promising for the discovery and testing of antitumoral treatments. They can be used as a tool for high-throughput drug screenings, for the identification of epigenetic or genetic causes of drug resistance, and for the comparison of different compound toxicities in normal versus tumoral tissues [10]. Therefore, the path has been paved for improving drug discovery and testing and avoiding the use of animal models. Here, we summarize the advances made in the establishment of cancer organoids in recent years.

#### Organoids of different types of cancer

- Colorectal cancer
  Colorectal cancer (CRC) is a heterogeneous disease with recurrent genetic mutations affecting mostly genes in five signaling pathways related to self-renewal and the proliferation of colon stem cells: Wnt, RAS/mitogen-activated protein kinase (MAPK), phosphoinositide 3-kinase (PI3K), TGF-β, and P53 signals [181]. The activation or inactivation of these pathways changes the niche microenvironment, enabling cancer cells to overgrow and even colonize other tissues; nevertheless, the interactions between cancer cells and their surroundings are still highly uncertain [119].
  Several studies have developed protocols and established CRC organoid and organoid biobanks [182, 183]. Fujii et al*.* generated 55 CRC organoid lines from a diverse range of CRC patient samples, including primary and metastatic lesions [184]. Researchers found that the concentration levels of Wnt activators (Wnt3A/R-spondin-1), the oxygen concentration and a p38 inhibitor (SB202190) are essential for CRC organoid proliferation. A representation of the PDOs was also xenotransplanted into the kidney subcapsules of immunodeficient mice, recapitulating the histological grading, morphology and differentiation patterns of the parental sites both in vitro and in the xenografted tumors. The resulting genetic profiles suggested that colorectal tumors are predominantly ruled by genetic mutations contributing to local tumorigenicity more than changes in tissue microenvironments. Correlation studies between genetic sequencing and drug sensitivity may find a gene-drug association that can be used in a personalized treatment approach; in this sense, it was found that PDOs with an E3 ubiquitin-protein ligase *RNF43* mutation, a negative Wnt feedback regulator, had a strong response to the small molecule porcupine (Wnt secretion) inhibitor IWP2 [182].
- Breast cancer
  In another approach with cancer organoids, Clevers and his team developed a breast cancer (BC) organoid protocol to generate long-term BC organoids from primary and metastatic samples, obtaining a living biobank of more than 100 genetically diverse BC organoid lines [166]. In this study, an optimized efficient protocol is described for the culture of BC organoids that involves the addition of Neuregulin 1, a ligand of human EGF receptor (HER) tyrosine kinases-3 and -4 implicated in mammary development and tumorigenesis, to the medium. Most BC organoids matched the BC of origin in terms of hormone and HER2 status and, with less efficiency, in histopathology. These BC organoids present a heterogenic phenotype without normal cells, enabling functional high-throughput drug screens.
  One of the great difficulties when establishing primary BC organoids is the presence of residual healthy cells from biopsies and their increased growth in 3D cultures compared to tumor cells. Goldhammer et al*.* evaluated the frequency of normal organoids in primary BC cultures and whether the cellular composition is stably maintained after several passages [184]. The high difficulty in establishing cell organoids from primary human breast cancer was noted due to the residual presence of healthy cells. Healthy organoids were present in all primary human BC-derived cultures and were more noticeable when passaged. In this sense, another study used a polymer scaffold and cancer-associated fibroblasts (CAFs) to culture primary BC organoids [185]. The protocol consisted of first culturing the CAFs in the scaffold to deposit specific ECM proteins, which helped in cell attachment and viability. Then, the cells were retired and primary BC cells were cultured in the scaffold. The use of an ECM secreted by CAFs from the patient enables us to obtain a more suitable microenvironment to grow primary BC organoids and capture intra- and interpatient tumor variability due to enhancement in cell–matrix interactions compared to bare scaffolds. Therefore, the platforms mentioned above could improve treatment, such as BC chemotherapy, and bring us closer to personalized medicine.
- Prostate and ovarian cancer
  In 2014, Gao and colleagues established prostate cancer organoid lines derived from patient prostate cancer metastasis samples with low long-term efficiency, but their efforts opened the door for the establishment of prostate tumoroids with different genotypic and mutation characteristics [186]. Later, Drost and colleagues developed a detailed protocol for prostate cancer organoids from human and mouse cells containing basal and luminal cells. They succeeded in culturing organoids from metastatic prostate cancer lesions and circulating tumor cells but not from primary tumors due to the higher proliferation rate of healthy cells [164].
  Ovarian cancer (OC) is a heterogeneous cancer usually diagnosed at a late stage when it has metastasized, and current in vitro models that represent its heterogeneity are limited. Current organoid models include a collection of 56 OC organoid lines of all the main subtypes derived from 32 patients, cultured long-term and cryopreserved with maintenance of intra- and interpatient heterogeneity [172]. OC organoid lines included both the primary tumor and the different metastatic lesions. Researchers based the method on a previous fallopian tube [177] protocol for medium optimization and observed that the addition of hydrocortisone, forskolin and heregulin β-1 and the withdrawal of Wnt-conditioned medium improved the formation of OC organoids with a success rate of 65%.
  Another publication of OC organoids by Phan et al*.* developed a method to cultivate organoids derived from two high-grade serous ovarian cancer patients and one ovarian carcinoma patient [187]. High-throughput drug screening was carried out within 1 week of surgery, which can help in predicting a therapeutic response before starting the treatment.
- Pancreatic and stomach cancer
  The late diagnosis and the lack of treatment options make pancreatic ductal adenocarcinoma (PDAC) one of the cancers with the worst survival rate. Current cytotoxic treatments do not work with many advanced patients, and unfortunately, there is not yet a personalized approach for treatment selection; thus, one of the best current options is early detection biomarkers for PDAC [188].
  Pancreatic organoids can be derived from tumor tissues and hPSCs [168, 169]. Clevers and Tuveson’s laboratory established mouse and human PDAC organoids. When orthotopically transplanted, PDAC organoids form early-grade tumors, as preinvasive pancreatic intraepithelial neoplasms, and progress to locally invasive and metastatic carcinomas [168]. Huang et al*.* established the conditions to generate primary human PDAC tumor organoid growth with matching of greater than 80% of the characteristics of the original tumors and identified changes in SOX9 location related to mutant TP53 expression in tumor organoids and differences in EZH2 among patients, which was frequently upregulated in patients with pancreatic cancer [169].
  Regarding stomach cancer, Yan et al*.* established a gastric cancer organoid biobank containing 7 normal and 46 gastric cancer organoid lines from different tumor regions and lymph node metastasis samples from 34 patients, with detailed whole-exome and transcriptome analyses [176]. Organoids were cocultured along with stromal cells to more closely resemble the tumor microenvironment. Some tumor subtypes with a strong tumor microenvironment resulted in single transcriptome signals and low tumor content.
- Head and neck cancer
  Head and neck squamous cell carcinoma (HNSCC) has no standard biomarker because of the lack of adequate tumor models. Tanaka et al*.* developed patient-derived HNSCC organoid models with the goal of characterizing them to improve the prediction of treatment strategies [189]. Patient-derived organoids were established with a rate of 30.2% retaining most histological features and properties of the original tumor. Additionally, organoids were compared with their derived 2D cell lines, which showed significant limitations, such as sensitivities to cisplatin and docetaxel. Later, Driehuis and colleagues developed a protocol for patient-derived HNSCC organoids embedded in basement membrane extract recapitulating the genetic and molecular characteristics of the original primary tumor and retaining tumorigenic potential [190]. Healthy oral mucosa-derived organoids were infected with human papillomavirus 16 (HPV16), which is known to contribute to the oncogenesis of a subset of HNSCC tumors, to validate the organoid model for this kind of mucosal pathology.
  Recently, HNSCC studies have focused on the search for more selective targeted therapies as an alternative for more personalized treatment. In this sense, EGFR-targeted photodynamic therapy has been successfully tested in patient-derived HNSCC organoids due to the overexpression of EGF in these tumors [191].
  Table 1 presents a summary of the different types of organoids, references to protocols establishing them, and the advantages/disadvantages of each system.

**Table 1:** Establishment of organoids will greatly benefit from a figure and/or table summarizing the different types of organoids, references to protocols of establishing them, advantages/disadvantages of each system etc.

Type of organoid	Cell source	Achievements	Protocol to establish them
Intestine	ASC (intestinal crypt Lgr5^+^ stem cells)	• Faithfully recapitulates the tissue • Long term growth • Growth of mouse adenomas, human colorectal cancer cells, and human metaplastic epithelia	[82, 84]
	iPSC	• The presence of mesenchymal cells leads to less niche factor requirements • Cost-effective	[85]
	ESC	• The epithelium contains functional enterocytes, as well as goblet, Paneth and enteroendocrine cells	[45]
Stomach	ASC (gastric epithelium Lgr5^+^ stem cells/TROY^+^ chief cells)	• Long-term growth • Robust numbers of surface pit, mucous neck, chief, endocrine and parietal cells	[87] [89, 90]
	PSC	• Primitive gastric gland- and pit-like domains, proliferative zones containing LGR5-expressing cells, surface and antral mucous cells, and diversity of gastric endocrine cells	[88]
Liver	ASC (Lgr5^+^ stem cells/ mature hepatocytes)	• Long-term growth • Cells can be converted into functional hepatocytes in vitro and upon transplantation into mice • Recapitulates the proliferative damage-response of hepatocytes	[35, 95]
	iPSC	• Cells in organoids differentiate into functional hepatocytes and cholangiocytes • The organoids organize a functional bile canaliculi system, which is disrupted by cholestasis-inducing drugs	[52]
	ESC	• Scalable culture system with a high level of recapitulation of the liver-specific microenvironment • Efficient hepatic maturation upon ex ovo transplantation	[229]
Pancreas	ASC (Lgr5^+^ stem cells)	• The organoid recapitulates the morphology, the epithelial polarization and the genomic stability of their origin tissue	[96]
	iPSC	• The organoids present an appropriate marker profile and ultrastructural, global gene expression and functional hallmarks of the human pancreas • Upon orthotopic transplantation into immunodeficient mice, these organoids form normal pancreatic ducts and acinar tissue resembling fetal human pancreas	[230]
	ESC	• Functional alpha, beta, delta, and polypeptide cells, and ability to secrete insulin-secretory granules	[76, 77]
Lung	ASC (basal cells, Clara cells and specialized alveolar type II cells (AEC2s))	• “Tracheospheres” derived from basal cells generate both secretory and ciliated cells • “Alveolospheres” derived from AEC2s cells contain both AEC2 and AEC1 present in the alveoli	[99, 100] [102]
	iPSC	• The organoids possess upper airway-like epithelium with basal cells and immature ciliated cells surrounded by smooth muscle and myofibroblasts as well as an alveolar-like domain with appropriate cell types • The cultures could be maintained for several months	[43, 53]
	ESC	• They include epithelial and mesenchymal cells and are able to produce surfactant	[43]
Brain	iPSC	• The organoids recapitulate progenitor zone organization, neurogenesis, gene expression, and a distinct human-specific outer radial glia cell layer • Co-culture of different parts of the brain can recreate the dorsal–ventral forebrain axis	[57, 60]
	ESC	• Generation of multiple organoids from different parts of the brain (midbrain, forebrain) • Electrically active and functionally mature neurons with dopamine production • In-vitro functional vasculature-like networks to increase the maturation of the organoid	[63, 78–80]
Retina	iPSC	• Retinal cups contain all major retinal cell types arranged in their proper layers • Their photoreceptors achieve advanced maturation, showing the beginning of outer-segment disc formation and photosensitivity	[115]
	ESC	• Fully stratified retinal tissue consisting of all major neural retinal components	[112, 113]

### Hereditary disease and gene therapy

Monogenic hereditary disease can benefit from organoid models to obtain more knowledge of the disease in specific organs and to develop possible treatments. Cystic fibrosis (CF) is a monogenic disease caused by a spectrum of mutations in the cystic fibrosis transmembrane conductance regulator (CFTR) gene, which mostly affects the pulmonary and gastrointestinal tract, causing the accumulation of viscous mucous. Dekkers and colleagues developed a powerful assay, termed forskolin-induced swelling (FIS), to measure CFTR activity [192] that is used in CF patient hiPSC-derived organoid studies [193]. Moreover, CF mutations may be corrected through CRISPR/Cas9-mediated gene editing technology. This technology has been used to correct the most common mutation, CFTRΔF508, in CF patient hiPSC-derived airway epithelial organoids without leaving any genomic scar [194] and shortly prior to that study to correct the same *CFTR* mutation in patient ASC-derived intestinal organoids [195]. However, given the multiorgan involvement of CF, it is still challenging to develop gene therapy.

In addition, CFTR functions as a bicarbonate (HCO^−3^) channel, which is essential for several cellular functions in the mucous epithelium and has long been ignored in current CF studies. HCO^−3^ efflux depends on both intracellular and extracellular chloride concentrations, which can switch CFTR permeability. The balance between the concentrations of both molecules is a key microenvironmental factor to take into consideration in future treatment research [196, 197].

On the other hand, there are several models of retinal organoids for the development of a gene therapy for retinitis pigmentosa, a retinal disease that remains incurable due to its extreme heterogeneity and unclear mechanisms. Several genes are involved, but the *RPGR* gene is one of the most prevalent. A study carried out in 2018 established patient iPSC-derived retinal organoids with *RPGR* mutations that were repaired via CRISPR/Cas9, resulting in repair of photoreceptor development and improvement in cilial length [198]. However, a similar approach with more patients presenting the same pathology would increase the possibility for future gene therapies.

### Drug discovery and personalized medicine

The current techniques used for drug screening rely on 2D cell lines that do not fully recapitulate the characteristics of real organisms and animal models, which are expensive and time-consuming and are unable to mimic the niche of cells in patients [199]. However, organoids have emerged as a potent tool to mimic organs in different developmental stages and disease states. Thus, the use of this model has become a promising approach in drug discovery and testing and in personalized medicine.

With this purpose, the development of organoid biobanks of different pathologies might have a huge impact on the drug development industry. Some of them are already emerging; for now, most of them consist of cancerous organoids. For instance, biobanks of glioblastoma [200, 201], hepatocellular [202], kidney [203] neuroendocrine [204], colorectal [182, 205, 206], pancreatic [207–209], prostate [210] and breast cancer [166] organoids have been described. There are still few biobanks for the study of other diseases, with one relevant one being that developed by Dekkers et al. in 2016 to investigate cystic fibrosis [210]. The development of biobanks representing more diseases would be an interesting course of action for the near future.

Some of these biobanks have already been used to test different drugs. For example, 83 compounds currently used in the clinic were screened in two colon organoid biobanks, one derived from healthy tissue and the other derived from cancer samples. They used them to test for known gene-drug associations and concluded that organoids can be used as a high-throughput drug screening platform [183]. The link between drug responses in patients and organoids derived from those patients’ metastatic biopsies has also been assessed, as well as their molecular profiling. Vlachogiannis et al. showed in their study that testing in gastrointestinal tumor organoids can serve as a guide for prescription in personalized cancer therapy [211].

Moreover, pancreatic patient-derived organoids (PDOs) have been used to compare their chemosensitivity with patient outcomes and to establish gene signatures to predict this response [212]. In an alternative approach, Huang et al. used primary human pancreatic ductal adenocarcinoma organoids to test the efficacy of various epigenetic regulators. They generated cancerous organoids by mutating the KRAS and p53 genes in normal pancreatic organoids, which are mutations that are frequently found in patients with this tumor type [169].

Chadwick et al. employed glioblastoma PDOs as a model to evaluate the usefulness of their newly generated 4D cell culture assays, defined as 3D models able to change in response to stimuli. Comparing the response of both models to different drugs, they concluded that 4D cell culture models can be used for high-throughput drug assessment [213]. Recently, PDOs were used to optimize the dose of sorafenib for patients with hepatocellular carcinoma, suggesting that this model might be useful for predicting patient-specific drug sensitivity to targeted drugs [214].

Additionally, a breast cancer biobank of 155 PDOs from breast cancer patients was generated and employed by Sachs et al. to compare the sensitivity to chemotherapy of patients and their respective PDOs, finding a correlation between their responses to tamoxifen [166]. Similarly, PDOs derived from ovarian, lung, head and neck, endometrium and bladder cancer have also been tested for chemotherapeutic drugs and targeted therapies, showing a promising correlation of their responses with patient outcomes [189, 214–218].

Interestingly, organoids can also serve as a platform to test drug toxicity, which is currently a major problem in clinical practice. They have already been proven to predict toxicity in some nontargeted tissues [219]. For instance, intestinal organoids have been shown to be an interesting system for testing drugs and toxins and determining the damage that they produce in the intestinal epithelium [220]. Moreover, hepatic organoids have been screened for drug-induced hepatotoxicity [221], kidney organoids for nephrotoxicity [54] and brain organoids for neurotoxicity [222], showing promising results.

Another significant challenge in which organoids can have a role is in the discovery of new biomarkers for different diseases. A biomarker, according to the FDA, is “a defined characteristic that is measured as an indicator of normal biological processes, pathogenic processes or responses to an exposure or intervention” [223]. They are widely used in cancer detection and stratification because they can be obtained through noninvasive techniques (e.g., blood extraction), and they can predict patient prognosis and sensitivity to therapy in multiple cases. Thirty potential tumor biomarkers have been identified by comparing healthy and primary liver cancer organoids [170]. New potential biomarkers have also been discovered for biliary tract carcinoma using organoids as a tool for genetic screening [224]. Another study using breast cancer organoids showed that the levels of DNA methyltransferases could be a biomarker for sensitivity to decitabine [225]. Recently, Gao et al. demonstrated that a glycan biomarker could be used to detect chemotherapy-resistant pancreatic ductal adenocarcinomas in clinical settings with the help of pancreatic organoids [226]. Altogether, these findings suggest a very promising future for organoids in the discovery of new hallmarks of cancer.

Immunotherapy has proven to be a potent tool for the treatment of tumors; however, a large proportion of patients cannot benefit from this therapy due to T cell and HLA heterogeneity. Fortunately, it has been shown that the generation of tumor-reactive T cells can be improved with the help of organoids [227]. In addition, the response to immune therapy can also be assessed by using this kind of model, as Della Corte CM et al. did with the combination therapy of anti-PD-L1 antibody with a MEK inhibitor (MEK-I) in non-small-cell lung cancer. This study suggests that the combination of both drugs is more effective than monotherapy [228].

At the moment, on the ClinicalTrials.gov website, there are over 80 ongoing and 2 completed clinical trials related to organoids. Even though most of the trials are focused on research related to cancer therapy and mechanisms of action (i.e., 69 clinical trials), some of them are centered on other pathologies, such as cystic fibrosis, inflammatory bowel disease, necrotizing enterocolitis, gut inflammation, diabetes, ciliopathy, infertility and spondylarthritis. Of the 2 completed studies, one was about cholangiocarcinoma, and the other was about cystic fibrosis. Notably, there were 9 studies in phase II and 2 studies in phase III. Collectively, these data suggest that organoids are not only being used as a preclinical model but are also being included in the clinical setting. This could entail a change in the paradigm of drug discovery, shifting from a collective to an individual approach to patients.

## Conclusions

There are multiple lines of evidence that organoids are an advancement for research and clinical applications, holding great potential to substitute or complement models currently used. They are being employed to achieve a better understanding of basic biology and to investigate numerous diseases. Because they are more representative than cell lines and less costly and time-consuming than animal models, they can be used for high-throughput drug screenings. In addition, PDOs are a remarkable option for personalized medicine, facilitating genetic screening and treatment testing. The ability of organoids to better recapitulate the microenvironment than previous models can help to shed some light on the interaction of cells with their niches and with elements of the immune system, both in normal tissues and in disease states (Fig. 3).

However, considerable research still needs to be performed to improve organoid systems. For instance, an important issue to address is standardization. Most protocols rely on the self-organization of stem cells; thus, there might be considerable batch-to-batch differences in organoids. Additionally, the fact that they are highly complex models thwarts their maintenance and tracking. It is vital to achieve a method to properly supplement them with nutrients and oxygen and to remove the unneeded substances. Hence, mimicking or recreating a vascular system should be a priority in the creation of organoids. This might allow them to grow larger and be maintained for a longer period of time and therefore to achieve more maturity in their developmental stage. Currently, their resemblance to fetal structures still hinders research on some processes.

In the future, the creation of assembloids (combinations or organoids) could serve as an instrument to deepen the understanding of the interaction between different organs and systems in our bodies. Tissue engineering might be useful to accomplish this type of approach. Moreover, emerging genetic manipulation tools, such as the CRISPR/Cas9 system, can employ organoids as a testing platform for modifications before translation to the clinic. This method has already been assayed in some pathologies, such as cystic fibrosis [194, 195] or retinitis pigmentosa [198], and could be useful in many other genetic diseases. Taking everything into consideration, there is still much to investigate in the field of organoids, but undoubtedly, they will become a considerable help in research and even clinical practice in the following years.

## Data Availability

All data used in this study are public.
